# Ferroptosis increases obesity: Crosstalk between adipocytes and the neuroimmune system

**DOI:** 10.3389/fimmu.2022.1049936

**Published:** 2022-11-21

**Authors:** Sen Zhang, Zhiyuan Sun, Xing Jiang, Zhimin Lu, Ling Ding, Chengzhi Li, Xuewen Tian, Qinglu Wang

**Affiliations:** College of Sport and Health, Shandong Sport University, Jinan, Shandong, China

**Keywords:** ferroptosis pathway, obesity, neurological dysfunction, inflammatory response, metabolic disorders

## Abstract

Ferroptosis requires not only the accumulation of iron ions, but also changes in many ferroptosis-related regulators, including a decrease in GPX4 and inhibition of SLC7A11 for classical ferroptosis, a deletion of FSP1 or GCH1. Surprisingly, adipose tissue (AT) in the obesity conditions is also accompanied by iron buildup, decreased GSH, and increased ROS. On the neurological side, the pro-inflammatory factor released by AT may have first caused ferroptosis in the vagus nerve by inhibiting of the NRF2-GPX4 pathway, resulting in disorders of the autonomic nervous system. On the immune side, obesity may cause M2 macrophages ferroptosis due to damage to iron-rich ATMs (MFe^hi^) and antioxidant ATMs (Mox), and lead to Treg cells ferroptosis through reductions in NRF2, GPX4, and GCH1 levels. At the same time, the reduction in GPX4 may also trigger the ferroptosis of B1 cells. In addition, some studies have also found the role of GPX4 in neutrophil autophagy, which is also worth pondering whether there is a connection with ferroptosis. In conclusion, this review summarizes the associations between neuroimmune regulation associated with obesity and ferroptosis, and on the basis of this, highlights their potential molecular mechanisms, proposing that ferroptosis in one or more cells in a multicellular tissue changes the fate of that tissue.

## Introduction

### Iron metabolism

Iron, which has the highest content of all essential trace elements in the human body, plays a critical role as a biologically essential component of every living organism (1), which is closely related to the development of the human body and the occurrence and development of diseases. Thus, a constant balance requires to be maintained between iron uptake, transport, storage, and usage to maintain iron homeostasis (2). Iron homeostasis involves the action of multiple cell types, including red blood cells, intestinal cells, hepatocytes, and macrophages (3). When the iron steady state is broken, its basic chemical properties lay the foundation for potential toxicity *via* the production of reactive oxygen species (ROS), including hydrogen peroxide (H_2_O_2_), hydroxyl radicals (·HO), superoxide anions (O_2_^−^), and other reaction intermediates with free radical properties (4, 5). During ATP production in mitochondria, some of the oxygen consumption will be reduced through the monovalent route (5), resulting in the production of the above reactive intermediates. Moreover, because of its redox properties, iron can just have a “Fenton reaction” with H_2_O_2_ in it, thereby generating extremely reactive ·HO. Furthermore, when ROS exceeds a certain threshold, it leads to oxidative stress (6, 7); therefore, too much iron can negatively impact the body. Increasing studies have proved that iron overload is the key cause of ferroptosis in cells, and the area of occurrence in the human body is extremely extensive (8). Additionally, long-term intake of a high-fat diet can lead to iron accumulation, which may exacerbate the mechanism of onset (9). The diseases associated with ferroptosis include neurodegenerative diseases (such as Alzheimer’s disease and Parkinson’s disease), ischemic reperfusion damage to the heart, brain, lungs, and kidneys, and stomach cancer and breast cancer (10–17).

### Mechanisms governing ferroptosis

**Figure 1:**
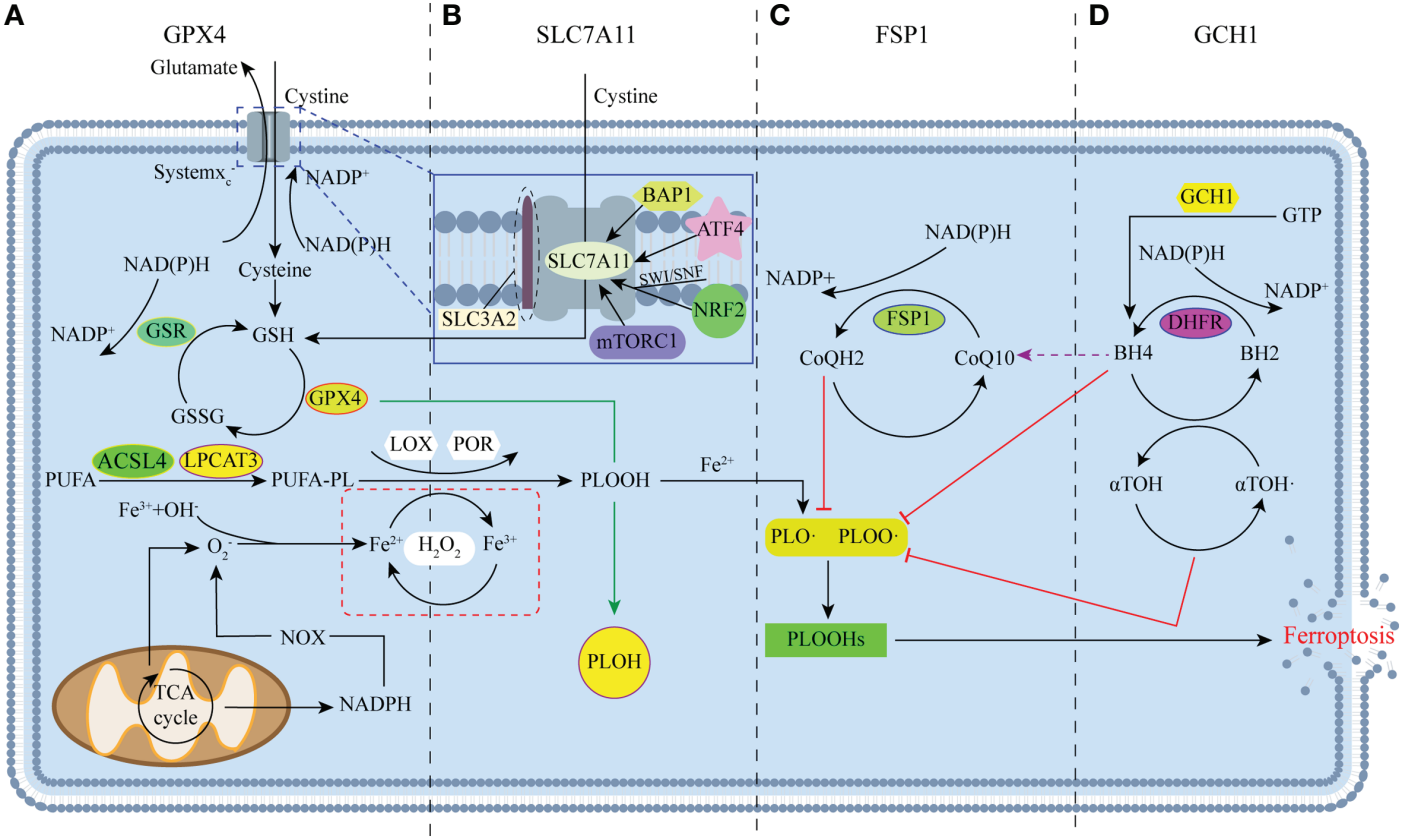
Major regulators of ferroptosis. **(A)** Glutathione peroxidase 4 (GPX4) is the key regulator of classical ferroptosis, and is mainly involved in the antioxidant axis system x_c_^-^-GSH-GPX4. **(B)** Many studies have focused on system x_c_-, with SLC7A11 acting as a mediator of system x_c_-. **(C)** FSP1 protects cells from ferroptosis through the NAD (P)H/FSP1/CoQ10 system as a result of genetic deletion or pharmacological inhibition of GPX4. **(D)** GTP cyclase hydrolase-1 (GCH1) protects cells from ferroptosis mainly through the GCH1/BH4/DHFR pathway.

Ferroptosis, unlike apoptosis, autophagy, and cell necrosis, is a regulatory form of cell death that depends on iron and ROS. This form of death was first reported in Brent R Stock well’s lab in 2012 (18). The mechanism of ferroptosis involves the highly expressed polyunsaturated fatty acids (PUFAs; such as docosahexaenoic acid and arachidonic acid) on the cell membrane, which are driven by acyl-Coenzyme A synthase long-chain family member 4 (ACSL4) and lysophosphatidylcholine acyltransferase 3 (LPCAT3). As a result, these PUFAs are modified to long chains and re-esterify them into phospholipids that infiltrate into lipids and membranes, forming PUFA-PL with carbon-centered phospholipid radicals (PL•) (19–22) (**Figure 1A**). Subsequently, non-enzymatic self-oxidation occurs with molecular oxygen under the catalysis of the Fenton reaction, which is initiated by iron and H_2_O_2_ to produce phospholipid peroxyl radicals (PLOO•), which are generated by removing hydrogen from another PUFA to generate PLOOH (6, 22). Simultaneously, with the participation of iron, PUFA-PL undergoes an enzymatic reaction to generate PLOOH under the direct or indirect catalysis of lipoxygenase (LOX) and cytochrome P450 oxidoreductase (POR) (23, 24) (**Figure 1A**). When PLOOH is not rapidly neutralized as an PLOH by glutathione peroxidase 4 (GPX4) in time, PLOOH further reacts with ferrous ions, and the resulting lipid radicals, such as alkoxyl phospholipid radicals (PLO•) and PLOO•, react with PUFA-PLs (8). By continuing the non-enzymatic reaction catalyzed by the previous removal of hydrogen atoms and the Fenton reaction with molecular oxygen, the production of phospholipid hydroperoxides (PLOOHs), known as ferroptosis executioners, continues. This ultimately generates numerous secondary products and various cascade peroxide chain reactions, as well as a large accumulation of lipid peroxides (such as malonaldehyde and 4-hydroxynenoic acid); these actions have the potential to destroy the integrity of the membrane, eventually leading to rupture or organelle membrane and cell death (25). During this process, O_2_^−^ produced by the mitochondrial tricarboxylic acid (TCA) cycle and the reduction of nitrogen oxides by NADPH produce H_2_O_2_ catalyzed by superoxide dismutase (SOD) and participate in the Fenton reaction of iron (26) (**Figure 1A**). Simultaneously, O_2_^−^ is also involved in the recovery of iron; PUFA removal of hydrogen can reduce trivalent iron ions to divalent iron ions, and trivalent iron ions can be reduced to divalent iron ions under the action of O_2_^−^ to generate molecular oxygen for PUFA-PL oxidation. The recovery between iron and ferrous is crucial for the Fenton reaction and lipid peroxidation process (24, 27).

This mechanism of death differs from that of other types of morphology, genetics, and biochemistry. Morphologically, ferroptosis is marked by a decrease or disappearance of mitochondrial crests, rupture and contraction of the outer mitochondrial membrane, and shrinkage and darkening of the volume and color of the mitochondria, but the morphological changes in the nucleus are not obvious (28, 29). In terms of genetics, ferroptosis leads to changes in iron transport pathways, RAS/MAPK/Raf pathways, and cysteine transport pathways, such as those including cyclooxygenase-2 (Cox2), ACSL4, and NADPH oxidases 1 (NOX1) genes, while also downregulating GPX4, solute carrier family 7 member 11 (SLC7A11), and Ferritin (30). In terms of biochemistry, ferroptosis occurs due a series of preparations, mainly manifested in the reduction of the antioxidant capacity of cells (18, 29, 31). For example, in the main inhibitory pathway of iron apoptosis, the system x_c_^–^GSH-GPX4 antioxidant axis, the upstream regulator system x_c_^-^ is inhibited, and the cystine transport pathway is blocked, resulting in a reduction in cystine transfer and cysteine reduced by NADPH (8, 25). As the rate-limiting precursor of glutathione (GSH), cysteine can cause a decrease in the activity of GPX4 and a large accumulation of lipid peroxides when deficient, which eventually leads to ferroptosis.

### Main regulator of ferroptosis

#### GPX4

Since Scott J. Dixon first discovered and named ferroptosis, the critical role of GPX4 in the inhibition of ferroptosis in his study has received widespread attention, mainly in the antioxidant axis system x_c_^-^-GSH-GPX4 (18). Due to GPX4’s excellent antioxidant capacity, it can detoxify intracellular lipid and cholesterol hydroperoxide product build-up by using GSH as a cofactor (32, 33). Furthermore, increasing studies have proven that GPX4 is the core regulator of ferroptosis (8). Indeed, as long as part of the GPX4 activity is maintained, cells lacking other selenoproteins can still survive and proliferate. However, the loss of GPX4 leads to the lethality of early embryos in mice. For example, in CAMKIIalpha-driven GPX4 knockout (KO) mice, mitochondrial dysfunction and ferroptosis due to chelated lipid peroxidation caused mice to display spinal motor neuron degeneration, paralysis, and death within 8 days of knockout (34), suggesting that cellular ferroptosis can be affected by regulating GPX4. GPX4 is also regulated in terms of activity and stability levels. GSH, as a cofactor is a direct factor affecting GPX4 (35), is derived from the tripeptides of glutamic acid, glycine, and cysteine, and is the most abundant reducing agent in mammalian cells (36). Notably, independent of the antioxidant properties of GSH, it also can regulate the NO steady state, forming nitrosyl mercaptans (37). In summary, GSH is important for the biological occurrence of iron-sulfur clusters, preventing oxidative damage, and directly regulating glucose homeostasis. When system x_c_^-^ is inhibited, obstruction of the cystine transport pathway can decrease GSH and GPX4, which can lead to cell death due to ongoing oxidative stress (**Figure 1A**).

In relation to obesity, genetic variants in GPX4 have been linked to obesity and inflammation in humans (38, 39), suggesting a role for GPX4 in metabolic disorders. And studies have described that impaired GPX4 activity is associated with obesity, although the biological consequences have not yet been determined (32). It is certain that obesity leads to the expansion of adipocytes, causing an inadequate supply of oxygen within AT and promoting the secretion of pro-inflammatory cytokines and ROS (40–42). The presence of the antioxidant GPX4 has also been shown to neutralize the ROS produced by the oxidative stress of AT and control AT inflammation by preventing lipid peroxidation (43). Furthermore, the same study also revealed that the activity of adipocytes GPX4 in obese patients is reduced, but the consequences seem to more of effect on undifferentiated adipocytes, but not as obvious for mature adipocytes (32, 43, 44). As a substance necessary for adipocytes differentiation, the reduction in GPX4 may affect AT remodeling, which is manifested by the finding that Gpx4^−/−^AT mice have larger adipocytes and a considerably increased body weight compared to wild-type mice; additionally, GPX4 can inhibits the phospholipid peroxidation of adipocytes, although the specific mechanism is unclear (43). Additionally, increased fasting blood glucose and insulin resistance were also observed in Gpx4^−/−^ AT mice, and spontaneously produced impaired insulin signaling in the liver, resulting in systemic inflammation and disorders of glucose metabolism (43). In conclusion, there is a strong association between GPX4 and obesity, and GPX4-centered ferroptosis may be the key link between GPX4 and obesity

#### FSP1

Ferroptosis-suppressor-protein 1 (FSP1) is a recently discovered non-GPX4-dependent ferroptosis inhibitory factor, which exhibits a different pathway of action from GPX4 in the process of ferroptosis inhibition, mainly regulated by the NAD (P)H/FSP1/CoQ10 system (45) (**Figure 1C**). As a consequence, both ultimately act on lipid radicals to reduce cellular damage caused by their excessive accumulation, and the loss of FSP1 can also increase PLOOHs. Apparently, FSP1 provides another important pathway for ferroptosis that is not limited to mitochondria (46, 47). Indeed, the sensitivity of different cancer cells to GPX4 inhibitors is highly variable, and some are unaffected by GPX4 (48). Therefore, the emergence of FSP1 may compensate for some of the shortcomings of GPX4. However, whether there is an FSP1-based pathway at play in adipocytes without ferroptosis when GPX4 is reduced requires further investigation. The available results suggest that FSP1 can play an important role in predicting the efficacy of ferroptosis-inducing drugs in cancer, and FSP1 inhibitors are considered to have great potential to overcome ferroptosis resistance.

#### GCH1

To identify other ways to regulate ferroptosis sensitivity, a new set of ferroptosis inhibition genes was identified by genome-wide activation library screening. As a result, a new ferroptosis inhibition axis with GTP cyclohydrolase-1 (GCH1) as the core was finally determined, which was found to be mainly regulated by the GCH1/BH4/DHFR pathway, which also protected cells from ferroptosis in a GPX4-independent manner (49, 50) (**Figure 1D**). However, studies only points to the key role of BH4 in protecting cells when GPX4 is inhibited, and its influence on lipid peroxidation is unrelated to its cofactors, but the specific mechanism remains unclear (50). Currently, the GCH1-based ferroptosis inhibition pathway only focuses on the therapeutic feasibility of tumors and cancer cells, with no obesity-related studies as of yet (49). However, as a polymorphism site for human and chronic diseases, including diabetes and hypertension, GCH1 can play a role in adipocytes and their related regulatory pathways.

#### SLC7A11 (xCT)

With the deepening of ferroptosis research, studies have strengthened the strategy of using the Cystine/Glutamate reverse transporter SLC7A11 (xCT) pathway to regulate ferroptosis, the principle of which is also based on the antioxidant axis system x_c_^-^-GSH-GPX4, but the focus falls on the key precursor system of the system x_c_^-^ (51). The system x_c_^-^ is actually a sodium-independent antiporter, consisting of the heavy chain subunit solute carrier family 3 member 2 (SLC3A2) and the light chain subunit SLC7A11 (52, 53). SLC3A2 can anchor SLC7A11 to the plasma membrane and maintain the stability of its protein (54), while SLC7A11 can mediate the activity of the reverse transporter of system x_c_^-^ (55, 56) (**Figure 1B**). The two work together to transfer extracellular cystine and intracellular glutamate out in a 1:1 ratio, thereby driving the efficient operation of the GPX4 antioxidant axis and protecting cells from oxidative stress damage (57, 58). However, because the function of SLC3A2 is not limited to cystine transport pathways, studies mainly focus on regulating SLC7A11 to inhibit the ferroptosis resistance of cancer cells (56). In addition, the activity and expression of SLC7A11 is strictly regulated by multiple mechanisms, such as transcription factor 4 (ATF4), nuclear factor erythroid 2–related factor 2 (NRF2), ubiquitin BRCA1 (Breast cancer gene 1)-associated protein-1 (BAP1), the SWI/SNF complex, and rapamycin complex 1 (mTORC1) (51), through which the expression of SLC7A11 can be upregulated to enhance the antioxidant defense of cells and inhibit the occurrence of ferroptosis (**Figure 1B**).

In general, there are four main mechanisms, GPX4, FSP1, GCH1 and SLC7A11, to help the body resist oxidative stress. Their operation is also precisely controlled by many regulators, but even under the conditions of so many pathways, more and more studies still regard SLC7A11 as the rate-limiting precursors and GPX4 as the core antioxidant defense to against ferroptosis (8).

## Association between obesity and ferroptosis

Studies found that obesity is closely related to iron metabolism disorders, which are mainly reflected as an excessive iron level (59). Ferroptosis caused by iron accumulation is accompanied by elevated ROS and inflammatory response, insulin resistance and mitochondrial dysfunction, leading to metabolic disorders and the development of obesity (60). As a result, the link between obesity and ferroptosis in different aspects has gradually become clear.

At the iron level, a previous study found that iron accumulation occurred in the epididymal adipose tissue (eAT) of polygenic obese and diabetic mice, but did not occur in the subcutaneous and brown adipose tissue (61). The available data are sufficient to show that iron accumulation is more obvious in the pancreas, liver, and heart, all of which are traditional tissues with excessive iron. Adipose tissue (AT) iron overload is also closely related to macrophages, mainly reflected in the important role of macrophages in controlling iron metabolism (62). Studies have shown that obesity induces increased M1 macrophages (M1) polarization in mice, which may further promote iron deposition, according to *in vitro* studies (63). Interestingly, studies have identified adipose tissue macrophages (ATMs) with iron-processing phenotypes, noting that a high-fat diet (HFD) promotes iron entry into adipocytes and inhibits the ability of ATMs to process iron (64). These studies also further illustrate the role of ATMs in the connection between iron and adipocytes. In addition, another study in mice with high-speed rail diet intervention also yielded an interesting result, showing that eAT produced robust remodeling, which was accompanied by AT weight reduction and a significant increase in iron content. This further suggests that processes associated with ferroptosis may play roles in AT remodeling (61). In a word, these findings further embody the relevant metabolic regulatory network in complex biological systems, with ferroptosis and obesity caused by iron accumulation as the connection point, and the parallel occurrence of the inflammatory response and insulin resistance.

Under an inflammatory environment and oxidative stress, as obesity progresses, the increased secretion of pro-inflammatory cytokines and chemokines, such as monocyte chemotin-1, tumor necrosis factor (TNF) α, and interleukin (IL)-6, lead to a low-grade inflammatory response throughout the body (65). This is similar to the environmental changes that accompany ferroptosis. Moreover, the increased iron is also accompanied by oxidative stress in the pancreas, liver, and eAT. In polygenic obese mice, iron chelating agents inhibit inflammatory factors and the production of oxidative stress, as well as the infiltration of ATMs to improve the hypertrophy of adipocytes (66, 67), which further indicates that ferroptosis may occur in AT. Indeed, the development of obesity and ferroptosis indicates the weakening of the body’s antioxidant capacity, which in turn leads to a series of metabolic disorders.

Regarding insulin resistance, studies have shown that omental adipocytes size and insulin resistance are positively correlated, and long-term obesity induces systemic inflammation and insulin resistance (68, 69). Moreover, an increase in inflammatory factors and a decrease in adiponectin may also lead to the interruption of insulin signaling pathways, reducing insulin sensitivity (70, 71). An increase in iron storage also causes insulin resistance, and insulin sensitivity increases after reducing serum iron levels. During insulin therapy, iron uptake can be increased by increasing the transferrin receptor 1 (TfR1) in adipocytes (72).

Regarding mitochondrial function, obesity can lead to mitochondrial dysfunction, an abnormality that mostly occurs in the liver, muscles, and AT that are associated with high energy, nutrient, and lipid overload (73). Furthermore, the morphology and number of mitochondria in obese patients have also changed. For example, the mitochondria in their skeletal muscle have become smaller and shorter, while those of white adipose tissue (WAT) are small and elongated, with an irregular crest, and the larger the adipocytes, the less mitochondria are present (74–76). Additionally, mitochondrial dysfunction in AT under obese conditions can significantly increase respiration and biogenesis, promote fatty acid oxidation and alter metabolism, all of which lead to the production of excess acetyl-CoA (77, 78). This is due to the fact that obesity enhances the production of ROS in mitochondrial, which can damage the lipid membranes, proteins, and enzymes of the respiratory chain in the mitochondria, as well as DNA (77). In summary, obesity leads to decreased mitochondrial function and increased insulin resistance. It is thought that there is a close link between the two given the known mitochondrial dysfunction caused by iron accumulation in adipocytes.

The special link between adipocytes and ferroptosis. Although there are substances, genes, and environmental changes within AT that are required for ferroptosis. However, there seems to be a contradictory result between adipocytes and ferroptosis, as shown by the proliferation of adipocytes despite being under ferroptosis conditions (79). Therefore, to demonstrate whether adipocytes can undergo apoptosis in response to oxidative stress, one study treated mature adipocytes with H_2_O_2_ and found that an increase in ROS enhanced adipocyte apoptosis (80). These results suggest that not only AT remodeling but also differentiated adipocytes can still be affected by ferroptosis. So how do adipocytes in AT defend against ferroptosis? One speculation is that although adipocytes have reduced GPX4, adipocytes may be similar to some tumor cells, which still have a high level of GSH in their cells, so that although adipocytes are in an overall oxidative stress condition, the remaining GPX4 from GSH is still needed to neutralize large amounts of ROS and remove some of the lipid peroxides, resulting in differentiated adipocytes that are not or less affected by ferroptosis (81). Compared to the former speculation, another study found that adipocytes can secrete specific fatty acids to induce ferroptosis resistance in breast cancer cells, and this process was dependent on the fatty acid synthetase ACSL3, confirming that breast cancer cells co-cultured with peri-tumor adipocytes showed resistance to ferroptosis (82). In addition, the team verified the protection of adipocytes against ferroptosis in triple-negative breast cancer through animal models. These results provide a new idea as to why adipocytes in obese AT do not undergo ferroptosis and continue to proliferate.

In conclusion, on the basis of these studies, this review speculated that there may be two mechanisms of ferroptosis leading to obesity: (i) In the autonomic nervous system, vagus and parasympathetic nerves in some organs or tissues may be affected by ferroptosis and become dysfunctional, while the inflammatory factors released from adipocytes lead to excessive sympathetic activation and hypothalamic neuronal ferroptosis which promotes the development of obesity through a series of processes; (ii) Ferroptosis may have occurred in certain anti-inflammatory immune cells in AT, which allows a large increase in pro-inflammatory immune cells, further causing systemic inflammation and insulin resistance, and ultimately leading to the development of obesity. Unfortunately, although ferroptosis, a novel mode of cell death, has received widespread attention from researchers since its discovery, there are still few studies on AT related to ferroptosis. Most of the studies are still mainly focused on tumor cells (50), and a certain theoretical system has not yet been formed, resulting in many specific mechanisms related to it are not very clear.

At present, the rapidly rising prevalence of obesity has become a global problem that needs to be solved. The discovery that AT can be affected by the process of ferroptosis provides us with another idea to treat obesity. Therefore, this review will investigate whether the neurological and immune systems can regulate the ferroptosis process in AT. Finding out the role of ferroptosis in AT, and thus provide a new therapeutic target for the treatment of obesity, which is important for the clinical treatment of human obesity.

## Neuromodulation of obesity and ferroptosis

**Figure 2:**
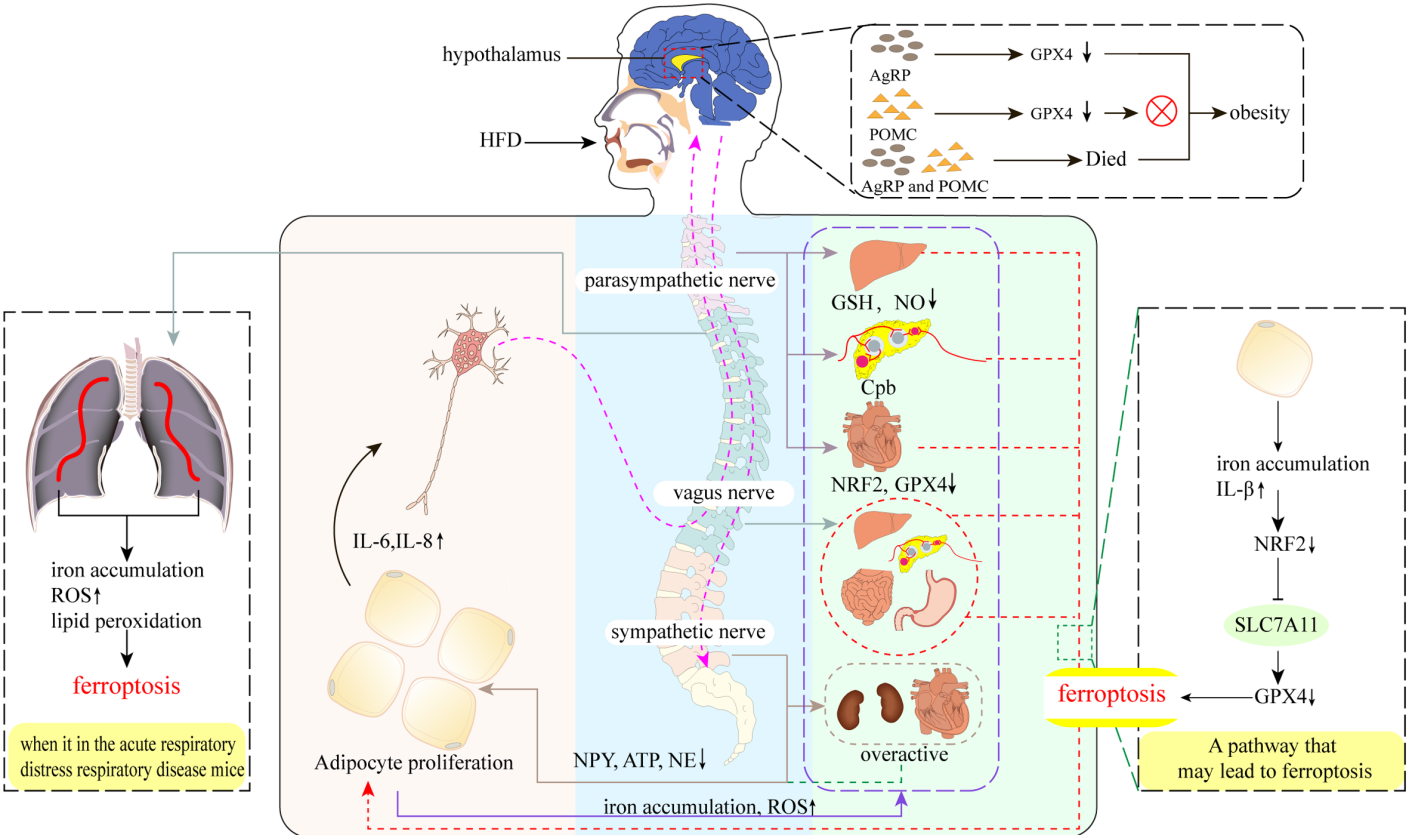
Neural regulation of obesity and ferroptosis. A high-fat diet (HFD) leads to increased secretion of pro-inflammatory cytokines such as IL-6 and IL-8 in adipose tissue (AT), which stimulates AT sensory nerves and subsequent vagal-parasympathetic transmission to the nociceptors, which then transmit regulatory information directly to AT *via* sympathetic nerves, as well as by targeting tissues and organs elsewhere in the body to promote AT lipolysis and thermogenesis. However, the emergence of obesity disrupts these transmission pathways. Parasympathetic: obesity decreases hepatic GSH, NO levels, pancreatic cholinergic pathway block (Cpd), and cardiac NRF2 and GPX4 levels, which may cause ferroptosis in hepatic, pancreatic, and cardiac parasympathetic nerves, thus promoting AT amplification. Vagus nerve: part of the parasympathetic nerve. The presence of obesity may cause hepatic vagal ferroptosis, which further leads to possible ferroptosis of islet and intestinal and gastric vagal nerves. Sympathetic nerves: obesity leads to over-activation of sympathetic nerves, including cardiac and renal sympathetic nerves, and reduced levels of the sympathetic transmitters NPY, ATP, and NE, which also reduce their direct innervation of AT. Hypothalamus: AgRP neurons do not undergo ferroptosis after the knockdown of GPX4, do not undergo ferroptosis after knockdown, yet still resulted in the appearance of obesity, which was not observed in POMC neurons. Interestingly, AgRP and POMC neurons also led to obesity after inactivation.

The nervous system is known to play a leading role in the regulation of physiological functional activities, but is also closely related to AT as a result of the dynamic crosstalk between adipocytes and other types of cells in the highly neurologically innervated and vascularized tissue matrix (83). Therefore, a large number of studies in recent years have actively explored the innervation of AT, and the results have confirmed the presence of only sensory innervation and sympathetic innervation within AT (83). Among them, the fat sensory nerve will convey the metabolic signal in AT to the central nervous system, and then instruct AT through the sympathetic nerve (84–86), so that it can carry out the corresponding lipolysis and thermogenesis according to the instruction (87, 88) (**Figure 2**). However, under obese conditions, the number of nerve fibers within the AT appears to be drastically reduced, which not only leads one to ponder whether there is some association with ferroptosis. Besides, the exact mechanism leading to the reduction of nerve fibers remains unclear. What is known is that the expansion of adipocytes causes tissue inflammation and diminished sympathetic signaling (83, 89). This leads to dysregulation of sympathetic neurotransmitter levels of NPY, ATP and NE, resulting in a chronic over-activation of sympathetic nerves, but with reduced sensitivity of β-adrenergic receptors, resulting in a weakened regulation of AT and a proliferation of adipocytes. Moreover, cytokines released from adipocytes such as leptin stimulate AT sensory nerve fibers and may lead to their hyperactivation and release of inflammatory factors, further inflammatory stimulation and oxidative stress may inhibit normal hypothalamic and nervous system function (89–91). In addition to the innervation within the AT, the autonomic nervous system is known to populate all organs and tissues of the body. As a whole, there is also a network of alternating influences between these autonomic nervous systems and the AT. For example, in the vagus nerve, although postganglionic neurons of the vagus nerve may be located outside of AT, studies have found that some preganglionic axons of the vagus nerve terminate on neurons of unknown phenotype in the adrenal, celiac, and superior mesenteric ganglia. Since neurons in these ganglia may contribute to sympathetic innervation of at least the intraperitoneal AT, this could also be a potential pathway for vagal innervation. Not only that, but studies has found ferroptosis in the nervous system (92). The reason may be related to the high activity of brain nerves. The high metabolic rate and high oxygen consumption of brain parenchyma will inevitably lead to the production of a large number of by-products ROS, and may further cause oxidative stress (93). At the same time, the brain is relatively limited in antioxidant factors, but it is also rich in iron, which can react with endogenous H_2_O_2_ to promote lipid peroxidation (94). And membranes with high PUFA-PL content should be particularly susceptible to peroxidation, as has been demonstrated in neurons (93). In addition to this, the iron accumulation seen in the liver of obese patients is an interesting echo of hepatic parasympathetic dysfunction. In conclusion, the systemic inflammation induced by obesity may accelerate the process of ferroptosis in certain neuronal cells, and this could potentially further promote the development of obesity (95).

## Vagus nerve

The vagus nerve is the main nerve of the parasympathetic branch (96), which can participate in taste sensation and innervate most organs, including the liver, stomach, and pancreas, and regulate circulation, breathing, and digestion by conducting sensory impulses in organs, particularly those controlling the activity of the heart muscle, glands, and smooth muscles (97, 98). In terms of the relationship with obesity, studies have found that in obese humans and rodent models, autonomic imbalances include excessive activation of the vagus nerve (99, 100), and a decrease in the mass of WAT is induced after vagus nerve resection (100). However, this idea, while demonstrating the ameliorative effect of vagus nerve excision on the association with obesity (101), is limited by the absence of significant changes in some lean animals and some populations (98), as well as the increased occurrence of secondary trauma and mortality after excision (102). As an important component of regulating metabolism and immune homeostasis, the vagus nerve can regulate WAT content through the central nervous system to control sympathetic peripheral muscle bundles, and can also regulate the release of GSH and NO to enhance antioxidant capacity and prevent ferroptosis (36). Thus, the vagus nerve may be involved in maintaining oxidative homeostasis and responding to external stimuli or injury. For example, in acute respiratory distress, ferroptosis has been found in mouse lung tissue, together with iron accumulation, increased ROS, and lipid peroxidation (103). The use of electro-acupuncture to stimulate the Zusanli (ST36) acupoint can activate the α7 nicotinic acetylcholine receptor in the lung tissue through the cervical vagus nerve and sciatic nerve to protect the ferroptosis in the lung tissue, which also verifies the importance of the vagus nerve in protecting the oxidative damage of cells (104) (**Figure 2**). The vagus nerve can also inhibit the release of pro-inflammatory cytokines in such a way that mediates selective cholinergic activation in the inflammatory response, thereby improving inflammation and obesity (97, 98, 105). Surprisingly, the emergence of obesity seems to break the protective ability of the vagus nerve, not only the oxidative damage of organs and tissues (i.e., the liver and pancreas), but also in terms of transmission signal interruption and decreased plasticity (98) (**Figure 2**). Indeed, some studies have pointed out that this phenomenon may be an important mechanism for developing obesity (106). Overall, autonomic imbalances caused by obesity may be associated with the first occurrence of ferroptosis in the vagus nerve, which impairs its ability to transmit information collected by the sensory nerve to the nerve center, as well as its ability to mobilize GSH and NO to prevent ferroptosis.

## Parasympathetic nerve

As another major component of the autonomic nervous system that neutralizes sympathetic activity, and has a complete mediated pathway to the pancreas, the parasympathetic nervous system plays an important role in maintaining metabolic homeostasis, such as maintaining blood sugar (107). Parasympathetic efferent nerves can transmit neuropeptides and acetylcholine to the islet liver, increasing insulin release and liver NO production through muscarinic receptors and lowering blood sugar. In addition, despite a lack of evidence of the parasympathetic innervation of WAT (108, 109), it can still affect WAT through multiple pathways, and studies have suggested that reduced parasympathetic activity is associated with obesity (110). Correspondingly, the link between parasympathetic injury and ferroptosis has gradually became clear. Diabetes mellitus, which is a complication of obesity, can activate satellite glial cells of the supracervical ganglia through P2Y receptors, resulting in increased expression of pro-inflammatory cytokines such as IL-1β, and causing oxidative damage to the autonomic nerves such as *via* an inflammatory response (111, 112) (**Figure 2**). Among them, oxidative damage to the autonomic nervous system due to iron accumulation associated with diabetes may be due to changes in the NRF2-GPX4 pathway in ferroptosis (113, 114), the transcription factor that regulates SLC7A11 (51) can effectively prevent oxidative damage in various pathological conditions upon NRF2 activation (115, 116). Furthermore, both obesity and diabetes are accompanied by significant increases in ROS and decreases in NRF2 and GPX4 levels, indicating a weakening of antioxidant capacity and activation of the ferroptosis process. Simultaneously, ferroptosis may be involved in the progression of cardiac autonomic neuropathy (DCAN) through the P2Y14 receptor (a new member of the metabolic receptor family), while P2Y14 targeting naringin can alleviate DCAN by promoting the NRF2-GPX4 pathway to eliminate ROS and inhibit ferroptosis (117) (**Figure 2**). Surprisingly, another study showed that diabetes-related cardiovascular autonomic damage is first manifested in the vagus nerve (106), which may involve a ferroptosis-mediated overall decrease in parasympathetic tone, ultimately leading to over-activation of the sympathetic nerve and the worsening development of metabolic complications. In conclusion, these studies elucidate the role of obesity and diabetes in promoting autonomic nervous system imbalances and metabolic disorders through the ferroptosis process (106).

## Sympathetic nerve

The sympathetic nervous system plays a key role in regulating metabolic control as an important hub that connects the brain to most organs, glands, and tissues, including the heart. Particularly in regulating AT, which is only innervated by the sympathetic nerves, while the liver, skeletal muscle, and pancreas are under the control of the parasympathetic nervous system (118). Studies have shown that sympathetic hyperactivation is strongly associated with hyperinsulinemia, impaired baroreceptors, angiotensin II, apnea syndrome, and adipokines (119). Furthermore, studies have highlighted that ROS can activate the sympathetic nervous system (120), suggesting that the sympathetic nervous system may be less inhibited under high oxidative stress. However, it has also been shown that sympathetic neurons undergo lipid peroxidation following cleavage of the p75 neurotrophic factor receptor, causing degeneration and cell death (121). Considered another way, sympathetic hyperactivation indicates that the inhibitory effects of the vagus and parasympathetic nerves have weakened, which may also be an important mechanism for developing obesity. In obese conditions, the vagus and parasympathetic nerves may be damaged due to ferroptosis, which also promotes further sympathetic activity and autonomic nervous system imbalance. Furthermore, studies have found that antioxidants can reverse sympathetic dysfunction, oxidative stress, and hypertension in male obese rats following intervention with antioxidants (122). It has also been shown that impaired baroreceptor reflexes are caused by reduced inhibition of the vagus and parasympathetic nerves (123). In conclusion, these data show that the overactivation of the sympathetic nerve under obese conditions is closely related to ferroptosis of the vagus nerve and the parasympathetic nervous system. Additionally, the sympathetic nervous system may have a complex mechanism that inhibits the effects of oxidative stress represented by ferroptosis.

## AgRP and POMC neurons

In addition to the vegetative nervous system described above, traces of ferroptosis have been found in neurons in some specific areas associated with obesity. For example, HFD has increased secretion of free fatty acids and pro-inflammatory cytokines. When these pro-inflammatory mediators reach the hypothalamus, they increased the levels of oxidative stress and neuronal inflammation in the hypothalamus through processes such as microglia proliferation (124, 125). Insufficient GSH, the only antioxidant in the hypothalamus, may cause ferroptosis in some neurons, leading to further metabolic disorders. Leptin-activated hypothalamic proopiomelanocortin (POMC) and agouti-related protein (AgRP) neurons located in the arcuate nucleus are typical (126, 127). The former increases appetite and the latter suppresses appetite, both of which are critical for the metabolic homeostasis of the body (128). Studies have shown that a high-fat, high-sucrose (HFHS) diet decreased GPX4 expression and activity in the hypothalamus of mice associated with elevated GPX4 levels in WAT (129). Surprisingly, the decrease in GPX4 failed to damage the integrity of both neurons, and mice that only lost GPX4 in AgRP neurons experienced weight gain, while POMC neurons required for ROS buffering and metabolic homeostasis were unaffected by GPX4 deletion (129) (**Figure 2**). This phenomenon is indeed elusive, but it is speculated that there may be other antioxidant pathways that inhibit this process. In contrast, another study that specifically induced the death of AgRP and POMC neurons, profound metabolic disorders and obesity emerged (129) (**Figure 2**). In summary, although the two neurons did not die of GPX4 reduction, the study focused on only one point of GPX4 and did not delve into other regulators, indicating that there are other pathways affecting the activity of POMC neurons under obesity conditions (129). However, this phenomenon also confirms that the increase in high levels of plasma under previous obesity conditions can cause POMC neuron stimulation, which will further induce sympathetic activation. Additionally, these studies continue to demonstrate that GPX4 deletion can cause oxidative damage to membranes and exacerbate neuroinflammation. Therefore, improving obesity by improving oxidative stress caused by ferroptosis in the hypothalamus and nervous system is an essential consideration.

## Immunomodulation with obesity and ferroptosis

**Figure 3:**
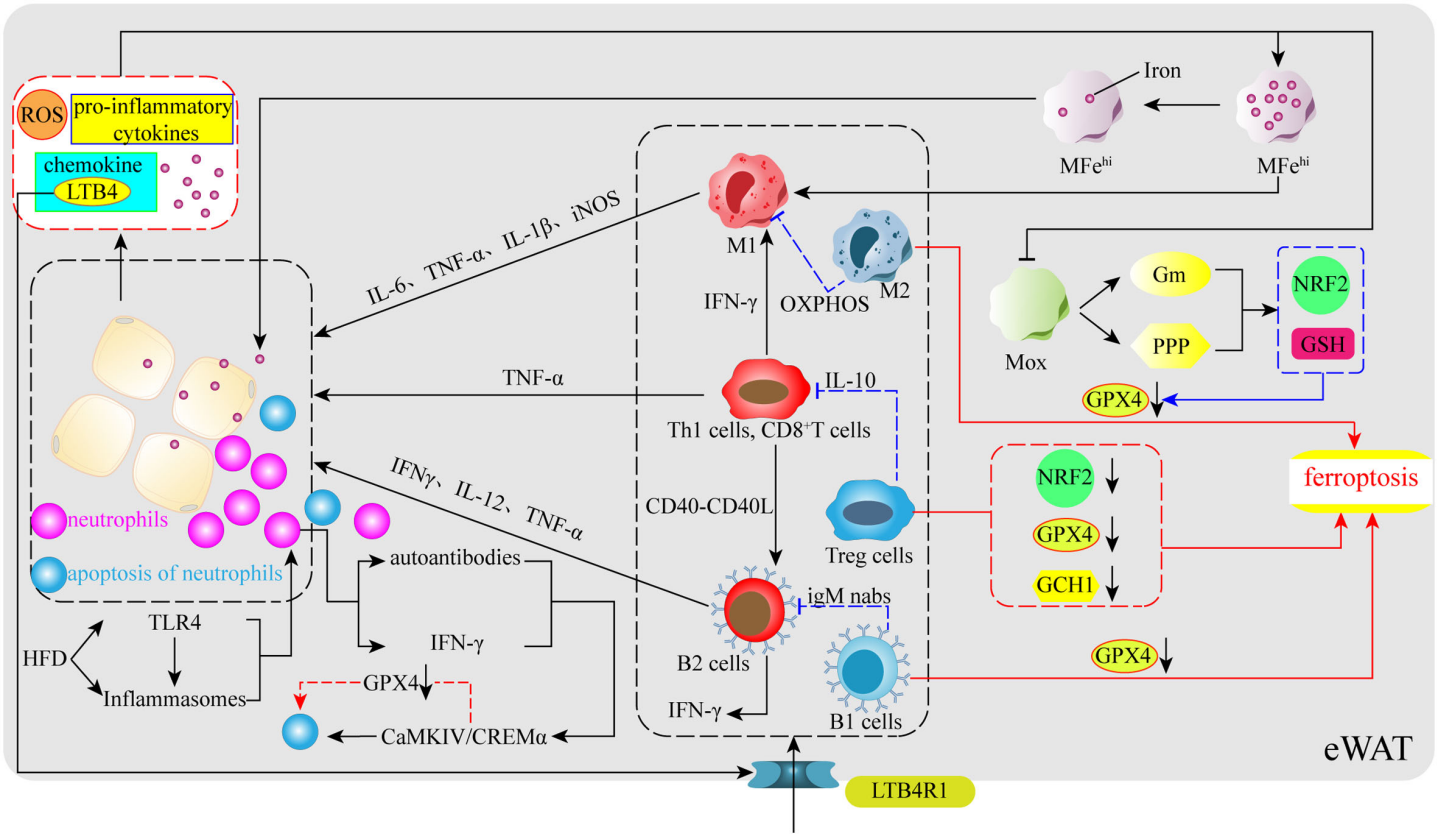
Immunoregulation of obesity and ferroptosis. In the early stages of obesity, HFD stimulates TLR4, a sensor within VAT, and inflammatory vesicles to release certain signals to promote neutrophil entry into VAT, followed by the release of chemokines (LTB4) by neutrophils to further promote the aggregation of ATMs, T cells, and B cells in VAT. Simultaneously, their pro-inflammatory phenotype releases numerous pro-inflammatory cytokines, which increases the levels of ROS, iron, pro-inflammatory cytokines, adipocyte-secreted chemokines, and leads to increased inflammation due to ferroptosis in some types of anti-inflammatory immune cells. ATMs: obesity impairs the iron reserve capacity of MFe^hi^ macrophages and promotes their polarization toward the M1 phenotype. At the same time, a large number of pro-inflammatory cytokines also inhibit the upregulation of the NRF2 gene and GSH levels through glucose metabolism and the pentose phosphate pathway in Mox macrophages, which have antioxidant capacity, resulting in reduced GPX4 levels and ferroptosis in M2 macrophages. T cells: Obesity causes T cells to release IFN-γ and CD40-CD40L to promote M1 and B2 production. Tregs undergo ferroptosis due to reduced levels of NRF2, GPX4, and GCH1. B cells: obesity promotes the entry of B cells into VAT *via* the LTB4R1 receptor, whereas B1 cells may also undergo ferroptosis due to reduced GPX4. Neutrophils: neutrophils activate the CaMKIV/CREMα axis to induce apoptosis spontaneously, which may involve ferroptosis-related processes.

With the continuous in-depth study of obesity, the importance of AT immune cells as a key hub for regulating the AT environment has become recognized. AT immune cells not only promote AT homeostasis, but also change phenotype and increase their number to promote the maintenance and development of local AT inflammation. AT immune cells also secrete pro-inflammatory cytokines and other pro-inflammatory products into the systemic circulation, which affect the entire body, thus representing a key factor in the transition from simple obesity to cardiovascular disease, type II diabetes, and related metabolic disorder complications (130). It should be clarified that although the contribution of immune cells to metabolic diseases is mainly concentrated in AT, an effective source of such inflammation also exists in the liver, intestines, and other metabolic regulatory tissues, promoting the production of inflammation and obesity throughout the body through the circulation (131). In addition, AT remains the most commonly studied source of immune-mediated inflammation in obesity. In the case of eWAT, an increase in endogenous fatty acids following adherence to a HFD activates two sensors within adipocytes, Toll-like receptor 4(TLR4) and inflammasomes, which causes an influx of neutrophils to enter the eWAT, leading to increased levels of chemokine and cytokine expression (132). Among them, the most typical is the chemokine Leukotriene B4 (LTB4), the increased expression of which causes recruitment of neutrophils, ATMs, T cells, and B cells, which increase expression of pro-inflammatory cytokines, eventually leading to the development of eWAT inflammation and obesity (132) (**Figure 3**). Therefore, in response to iron accumulation and high levels of ROS within AT, certain anti-inflammatory immune cells may also be affected by the ferroptosis process, resulting in their inability to neutralize the inflammatory response caused by pro-inflammatory cytokines in a timely manner. Currently, known immune cells present within AT include ATMs, T cells (CD4^+^, CD8^+^, Tregs), natural killer T cells, B cells, neutrophils, eosinophils, and dendritic and mast cells (130, 133, 134). Based on current research progress and the association of these immune cells with obesity and oxidative stress, this review identified macrophages, T cells, B cells, and neutrophils, which are closely related to oxidative stress and metabolic disorders, and sorted out their changes in obesity and these immune cells complication-related pathways to identify ferroptosis traces.

## Macrophages

ATMs are central players in obesity-related inflammatory and metabolic diseases and can be involved in the regulation of adipocytes energy metabolism and mitochondrial function (133), thereby promoting post-inflammatory or post-injury repair, as well as tissue remodeling (135). ATMs have two main phenotypes: M1 macrophages (M1) is considered the culprit of AT inflammation, can participate in glycolysis to rapidly produce ATP, and plays a key role in the initiation and maintenance of systemic inflammation and insulin resistance; and M2 macrophages (M2) uses the effective coupling of respiration in the electron transport chain to enhance oxidative phosphorylation (OXPHOS), reduces ROS production, and promotes improved obesity and systemic glucose homeostasis by inhibiting adipocytes proliferation (136, 137). Additionally, studies have identified two new ATMs that are closely related to iron metabolism and oxidative stress: iron-rich ATMs (MFe^hi^) and antioxidant ATMs (Mox) (138). Impaired MFe^hi^ affects iron distribution, leading to AT dysfunction and adipocyte iron overload in obese patients; Mox inhibits oxidative damage by upregulating NRF2-dependent antioxidant enzymes and producing GSH, which has been shown to be the main ATMs for lean AT (64, 138).

At present, the interaction between ATMs and ferroptosis is gradually becoming clear, studies have shown that ATMs secrete IL-6, TNF-α, IL-1β, and iNOS, which can induce or inhibit the process of ferroptosis through different routes (139–142). Notably, IL-6 not only modulates hepcidin to affect the release of stored iron from ATMs and intestinal absorption, but also reduces transferrin expression and aggravates iron accumulation by promoting hepcidin production. IL-6 can even induce the polarization of ATMs to M1 or M2 (143). IL-1β can also activate hepcidin to promote transferrin degradation and iron accumulation, which is also related to the occurrence of ferroptosis (144). In addition, the ROS produced by ATMs first affect their recruitment and polarization, especially in cases such as diabetes, ROS induces ATMs to polarize towards M1 and the release of inflammatory factors (145, 146). Based on these results, an interesting association emerged, in that obesity leads to the accumulation of ATMs; indeed, in most obese individuals, up to half of the cells in the fat pool are ATMs (147). However, iron accumulation still occurs in AT in obese patients, which indicates that IL-6 and IL-1β in AT may cause induce damage to MFe^hi^ and Mox, resulting in high levels of oxidative stress and lipid peroxidation of AT (64) (**Figure 3**). Furthermore, as HFD mice show a decrease in M2 marker expression, does the inflammatory environment and iron accumulation cause M2 ferroptosis? Recent reports have found that GPX4 deletion induces iron apoptosis in M2, while M1 does not, largely because iNOS is a negative regulator of iron apoptosis and promotes M1 survival (148). In addition, another study showed that ATMs taken from the bone marrow died of iron in M0 in the absence of GPX4 (148). As the original source of ATMs, this phenomenon also provides a certain basis for the possibility of ferroptosis of ATMs. In short, AT under obese conditions due to the presence of many pro-inflammatory factors and iron accumulation, which may trigger the occurrence of ferroptosis and inflammation in M2, eventually leads to high rates of adipocytes proliferation and further development of obesity.

## T cells

Following the identification of macrophages as the culprit of most inflammatory events, studies have found that when the obese phenotype is activated, T cells are involved in the inflammatory response (149, 150). However, more T cells located in visceral adipose tissue (VAT) play a role compared to those in subcutaneous fat; this is because VAT contains more immune cells compared to subcutaneous fat and the main immune cell types in VAT are ATMs and T cells, which play a more critical role in immune metabolic homeostasis (151). Similar to ATMs, T cells have both pro- and anti-inflammatory action types; indeed, Th1 cells have pro-inflammatory effects (a CD4^+^ T cell subsets) as do CD8^+^ T cells, while Th2 cells and regulatory T cells (Tregs) have anti-inflammatory effects (152). Tregs are mainly derived from the thymus gland or peripheral gland and serve to regulate local and systemic inflammation and metabolism. However, studies have shown that Tregs in HFD mice VAT decrease with age, and Treg cells are also significantly reduced in obese patients (153, 154). Interestingly, another study mentioned the key role of GPX4 in preventing ferroptosis in activated T cells (155). Thus, under obese conditions, inhibition of antioxidant enzyme pathways may cause ferroptosis in activated Treg cells, decreasing their number and increasing inflammation and metabolic disorders (156). Surprisingly, according to the previous system, the obesity-induced oxidative stress should lead to ferroptosis of traditional T cells, but the number of CD4^+^ T cells with pro-inflammatory effects and CD8^+^ T cells has been found to increased significantly (152). However, there are currently fewer mechanisms for regulating ROS in T cells, so it is still difficult to explain how the altered systemic metabolic environment after obesity affects T cells in the context of the immune response. However, another study demonstrated that NRF2 is a key factor in facilitating Treg resistance to ROS (157, 158). HFD inhibits the transcription of key antioxidant genes by triggering NRF2 translocation into obese mouse Tregs nucleus, causing a rapid decline in HO-1 expression in Tregs, leading to enhanced oxidative stress and possible ferroptosis, and causing an intensification of obesity and inflammatory responses (156) (**Figure 3**). Additionally, studies have pointed to the effect of BH4 inhibiting ferroptosis in T cells, and when the rate-limiting enzyme GCH1 of BH4 is deficient, it will inhibit the proliferation of T cells (159) (**Figure 3**). In summary, a large decrease in Tregs under obese conditions may be greatly related to ferroptosis, which may also fall on the classic antioxidant axis x_c_^−^-GSH-GPX4, serving to induce ferroptosis in activated Tregs in this antioxidant pathway. Simultaneously, GPX4 is essential for the maintenance, immunity, and proliferation of regular T cells.

## B cells

Recently, studies have shown that B cells are also involved in the regulation of obesity-induced AT inflammation and insulin resistance (131, 160–162). As effector cells that produce specific antibodies, B cells can be mainly divided into B1 and B2 cells; among which, B2 cells have a pro-inflammatory effect. In an obese environment, many B2 cells are recruited into VAT through the action of the chemokine leukotriene B4 (LTB4) and its receptor LTB4R1, leading to tissue inflammation and the development of insulin resistance (163) (**Figure 3**). For B1 cells, B-1b cell subsets and the anti-inflammatory IgM natural antibodies alleviate dietary glucose intolerance and obesity metabolic dysfunction (164). However, among the pro-inflammatory and anti-inflammatory B cells, obesity is largely characterized by B2 cell accumulation and B1 cell damage within VAT (163, 165). Interestingly, GPX4 prevents ferroptosis due to lipid peroxidation in B1 cells, but is optional for maintenance and development in B2 cells (166). Therefore, it is important to determine whether the high concentration of ROS and the decline in antioxidant capacity caused by obesity causes ferroptosis in B1 cells. This is currently uncertain, with many unexplained mechanisms between AT, GPX4, and immune cells. It is certain that while B1 cells from the spleen did not die of iron during GPX4 deficiency, B1 cells taken from the abdominal cavity of mice experienced a higher degree of ferroptosis at the time of GPX4 defect (166) (**Figure 3**). It is worth pondering whether B1 cells in the obesity eAT will also undergo similar changes.

## Neutrophils

New research data suggest that neutrophils are also involved in the development of AT inflammation and insulin resistance, for instance, by infiltrating AT early in obesity and producing chemokines and cytokines that promote ATM infiltration (167). Neutrophil-derived proteolytic enzymes (i.e., myeloperoxidase and elastase) may also be involved in the initiation of AT inflammation (132). On this basis, some studies have indicated that an HFD caused by the NF-kB pathway increases the specific expression of IL-1b in eWAT neutrophils, which far exceeds the contribution of ATMs (132). Taken together, these results support the idea that neutrophils penetrate AT, causing high numbers of ATMs and IL-1b levels to drive obesity and insulin resistance (132). Surprisingly, to prevent activated neutrophils from releasing their highly cytotoxic inflammatory mediators into the tissue environment and damaging surrounding tissues along the bloodstream, neutrophils spontaneously undergo apoptosis. Although the mechanism that triggers this apoptosis remains unclear, the apoptosis process plays a key role in maintaining immune homeostasis and eliminating inflammation (168). Furthermore, neutrophil apoptosis is accompanied by a lack of GSH, and GSH may play an important role in this apoptosis process. Correspondingly, treatment with exogenous GSH and LPS was found to delay apoptosis and reduce the level of the pro-apoptotic protein caspase-3 (169). Although the relevant data do not prove the effect of ferroptosis in this process, the effect of GSH infers an association with ferroptosis. Therefore, to explore whether there is an association between neutrophils and ferroptosis, another study explored the role of GPX4 in neutrophil ferroptosis and identified two key factors, namely serum autoantibodies and IFN-α (170), both of which induce iron apoptosis in neutrophils by activating the CaMKIV/CREMα axis, leading to SLE neutropenia. An increase in the nuclear translocation of CaMKIV/CREMα decreases GPX4, which is accompanied by an increase in intracellular lipid-ROS, eventually leading to neutrophil iron apoptosis (170) (**Figure 3**). Although the focus of the study was on systemic lupus erythematosus, the data demonstrate that ferroptosis can occur in neutrophils (170). Overall, these data suggest a strong link between neutrophils and ferroptosis. The accumulation of many neutrophils in obese AT shows that neutrophils are unaffected by ferroptosis, which may be similar to other pro-inflammatory immune cells. However, the role of neutrophil ferroptosis in immunodeficient diseases is worth considering, particularly whether there are shared pathways that mediate the process of ferroptosis.

## Conclusions and outlook

**Figure 4:**
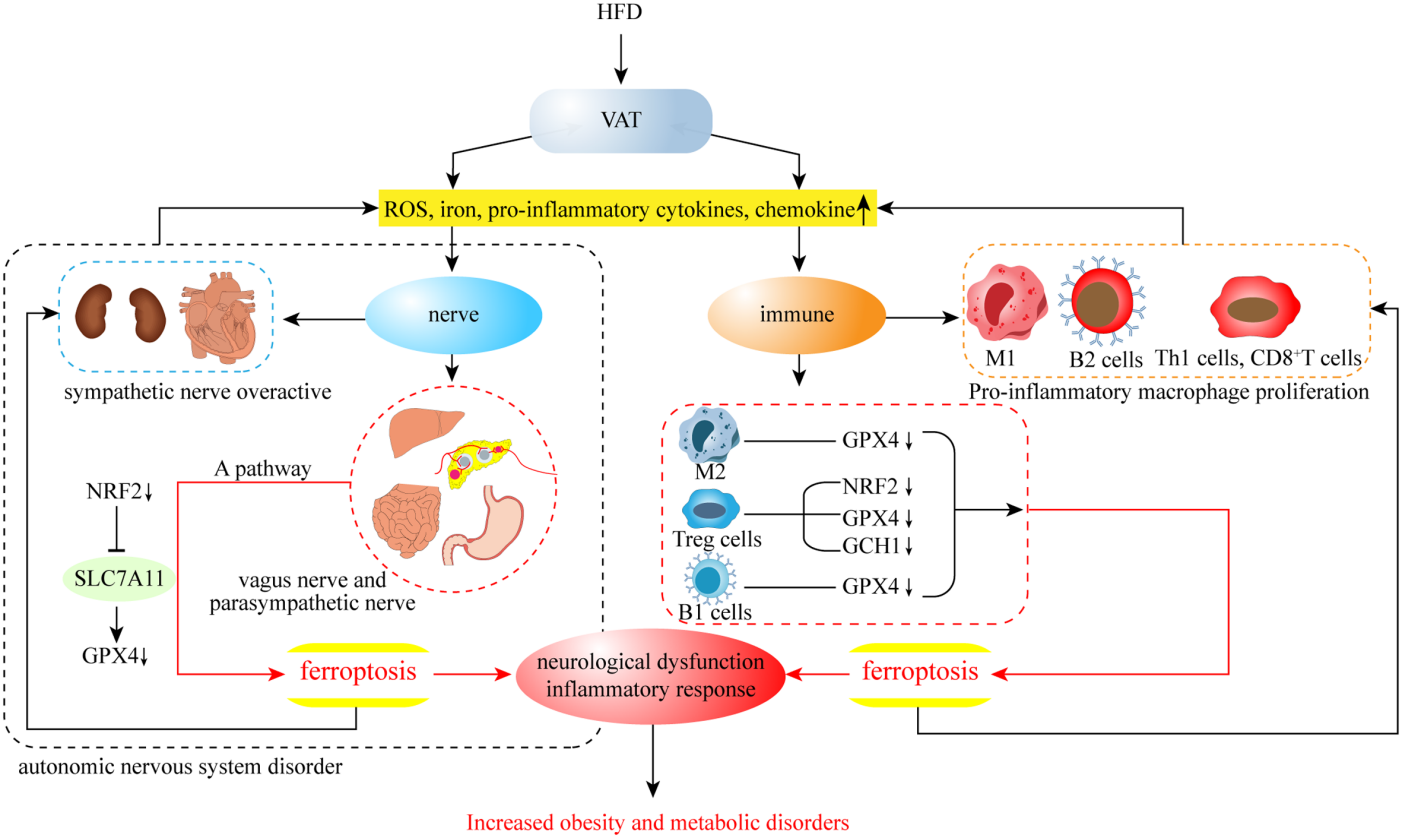
In VAT, HFD leads to an increase in ferroptosis-related factors, which may further contribute to the development of obesity from both neurological and immune aspects.

This review summarizes the characteristics of iron metabolism in humans, the association between obesity and ferroptosis, and the neuroimmune regulation associated with obesity and ferroptosis. While many of these mechanisms are still unknown, there is growing evidence that ferroptosis plays a key role in obesity and its complications, including type II diabetes, hypertension, nonalcoholic steatohepatitis, atherosclerosis, and obese cardiomyopathy (171). Moreover, ferroptosis may not only play a direct role in AT, but also indirectly promote the development of obesity through the inflammatory response and insulin resistance caused by liver autonomic and immunomodulatory disorders(**Figure 4**). Indeed, ferroptosis occurs through the intersection of multiple pathways to form an intricate regulatory network with obesity, so that one or more cells in the AT ferroptosis has the potential to change the fate of the entire tissue development. Therefore, research related to obesity with different ferroptosis targets is constantly emerging, and its specific mechanisms are gradually becoming clear. In addition to this, research into cuprotosis, which is similar to ferroptosis, has also appeared in recent years. As the name suggests, this is a form of cell death caused by copper, in which copper ions directly bind to protein lipoylation components in the tricarboxylic acid circulation pathway, causing abnormal aggregation of lipoylated proteins and loss of iron-sulfur cluster protein, resulting in protein toxic stress responses and cell death (172). Interestingly, the cuprotosis approach to cell death caused by the tricarboxylic acid cycle is similar to ferroptosis, in that ferroptosis also involves mitochondrial dysfunction and the production of excess acetyl-CoA. Thus, it is worth exploring whether the development of cuprotosis is indirectly related to ferroptosis. Ultimately, the current research on both ferroptosis and cuprotosis is still in its infancy, and it is not yet possible to confirm their specific mechanisms. However, the performance of nerve and immune cells involved in their action process still proposes novel therapeutic targets for obesity and related metabolic diseases, as well as cancer. With the continuation of research, the mechanism of ferroptosis and obesity metabolic disorders will likely be deciphered in the near future, opening up the option to reasonably mediate cellular ferroptosis through accurate scientific targets for treating obesity.

## Author contributions

SZ read the literature related to the topic and participated in drafting the manuscript. ZS, XJ and ZL participated in searching and archiving the literature related to the topic and discussed the contents of the manuscript. LD, CL, QW and XT revised the manuscript. All authors contributed to the article and approved the submitted version.

## Funding

This work was supported by grants from the Taishan Scholars Program of Shandong Province (tsqn201909148), the central government guides local science and technology development funds (YDZX2022091), and the technology research and integrated application of scientific fitness smart chip and cloud service platform(2020CXGC010902).

## Acknowledgments

We thank LetPub (www.letpub.com) for its linguistic assistance during the preparation of this manuscript.

## Conflict of interest

The authors declare that the research was conducted in the absence of any commercial or financial relationships that could be construed as a potential conflict of interest.

## Publisher’s note

All claims expressed in this article are solely those of the authors and do not necessarily represent those of their affiliated organizations, or those of the publisher, the editors and the reviewers. Any product that may be evaluated in this article, or claim that may be made by its manufacturer, is not guaranteed or endorsed by the publisher.
